# Neural correlates of control over pain in fibromyalgia patients

**DOI:** 10.1016/j.nicl.2023.103355

**Published:** 2023-02-21

**Authors:** Benjamin Mosch, Verena Hagena, Stephan Herpertz, Michaela Ruttorf, Martin Diers

**Affiliations:** a Department of Psychosomatic Medicine and Psychotherapy, LWL University Hospital, Ruhr University Bochum, Bochum 44791, Germany; b Computer Assisted Clinical Medicine, Medical Faculty Mannheim, Heidelberg University, Mannheim 68167, Germany; c Mannheim Institute for Intelligent Systems in Medicine, Heidelberg University, Mannheim 68167, Germany

**Keywords:** Fibromyalgia, Pain, fMRI, Functional connectivity, Voxel-based morphometry

## Abstract

The perceived lack of control over the experience of pain is arguably-one major cause of agony and impaired life quality in patients with chronic pain disorders as fibromyalgia (FM). The way perceived control affects subjective pain as well as the underlying neural mechanisms have so far not been investigated in chronic pain. We used functional magnetic resonance imaging (fMRI) to examine the neural correlates of self-controlled compared to computer-controlled heat pain in healthy controls (HC, n = 21) and FM patients (n = 23). Contrary to HC, FM failed to activate brain areas usually involved in pain modulation as well as reappraisal processes (right ventrolateral (VLPFC), dorsolateral prefrontal cortex (DLPFC) and dorsal anterior cingulate cortex (dACC)). Computer-controlled (compared to self-controlled) heat revealed significant activations of the orbitofrontal cortex (OFC) in HC, whereas FM activated structures that are typically involved in neural emotion processing (amygdala, parahippocampal gyrus). Additionally, FM displayed disrupted functional connectivity (FC) of the VLPFC, DLPFC and dACC with somatosensory and pain (inhibition)-related areas during self-controlled heat stimulation as well as significantly decreased gray matter (GM) volumes compared to HC in DLPFC and dACC. The described functional and structural changes provide evidence for far-reaching impairments concerning pain-modulatory processes in FM. Our investigation represents a first demonstration of dysfunctional neural pain modulation through experienced control in FM according to the extensive functional and structural changes in relevant sensory, limbic and associative brain areas. These areas may be targeted in clinical pain therapeutic methods involving TMS, neurofeedback or cognitive behavioral trainings.

## Highlights

• Healthy controls activate prefrontal & cingular cortices during controlled pain.
• Patients with fibromyalgia (FM) fail to activate these brain regions.
• Uncontrollable pain leads to orbitofrontal activations in healthy controls.
• In FM, uncontrollable pain leads to limbic activations.
• Decreased functional connectivity & gray matter volume of pain-modulatory areas in FM.

## 1 Introduction

It is commonly known that cognitive and emotional variables can attenuate the experience of pain. Relevant pain-modulatory factors are e.g. attention, anticipation, catastrophizing and (re-)appraisal. Behavioral data shows that acute pain tends to be perceived as less intense when the noxious stimulation can be terminated by oneself and thus appears to be controllable (Borckardt et al., 2011, Mohr et al., 2012, Müller, 2011, Weisenberg et al., 1985, Wiech et al., 2006). It has been proposed that perceived control evokes a form of cognitive re-interpretation (“reappraisal”) of the painful stimulus, ultimately making the associated experience less threatening (Skinner and Zimmer-Gembeck, 2010).

On a neural level, a number of areas implicated in voluntary reappraisal have been shown to play a pivotal role in pain modulation through perceived control. Current research focusses primarily on the prefrontal cortex (PFC). With regard to previous investigations by Wiech et al. (2006 & 2014), the ventrolateral prefrontal cortex (VLPFC) in particular appears to be of great significance for the processes described above. Furthermore, the dorsolateral prefrontal cortex (DLPFC) has repeatedly been shown to be a crucial part of the descending pain control network and evidentially plays a critical role in top-down modulation of the painful experience (Krummenacher et al., 2010, Tracey and Mantyh, 2007). The area has also been shown to be involved in analgesic responses due to religious beliefs (Wiech et al., 2008) and the administration of placebo treatments (Krummenacher et al., 2010).

With the uncontrollability of repetitive pain attacks being a major source of sorrow and agony in chronic pain patients, the effect of perceived control on the experience of pain is a particularly important aspect to consider when investigating chronic pain populations. The precise way in which controllability affects the subjective experience of pain as well as the underlying neural mechanisms, have thus far mainly been explored in healthy controls (HC) and are yet to be investigated in chronic pain patients. However, understanding the brain mechanisms involved in processing of controllable pain and potential disturbances in patients with chronic pain disorders such as fibromyalgia (FM) could help improve treatments. In this respect, we chose FM patients as a target group, as FM has repeatedly been shown to be associated to disrupted pain processing (e.g., Gracely et al., 2002). The goal of our investigation was to explore the modulation of experimental pain through the experience of control in HC and FM.

For HC, we assumed that perceived controllability of heat pain would lead to neural activity in pain regulation and voluntary reappraisal-related brain areas that were used as regions of interest (ROIs), as the VLPFC, (right) DLPFC and dorsal anterior cingulate cortex (dACC) (Kalisch et al., 2006, Mohr et al., 2012, Wiech et al., 2006). The corresponding findings were compared to FM. In order to obtain a comprehensive picture of the disorder-specific neural changes in FM, we additionally assessed functional connectivity (FC) and brain morphometric GM changes of the aforementioned brain areas.

## 2 Materials and methods

### 2.1 Participants

22 female FM (aged 50.48 ± 9.89 years, range 32 to 68 years) and 21 female HC subjects (aged 46.62 ± 13.08 years, range 25 to 68 years) participated in the study. Age was not significantly different between groups (*t*(42) = 1.1, p =.273) and there was no missing data. Left handed persons (Oldfield, 1971) were excluded. All participants had normal or corrected-to-normal vision. FM diagnoses were obtained by medical professionals and disorders fulfilled the criteria postulated by Wolfe et al. (2016) (see Table S1 also for additional demographic, psychometric and clinical data). FM reported a mean pain duration of 14.88 years (SD = 11.82; range 2 to 44 years) and were mainly recruited through social media support groups. HC were recruited via newspaper announcements and face-to-face acquisition at blood donation events of the German Red Cross. None of the tested participants had taken pain medication on the examination day, opioid use had been suspended no later than 3 days prior to the magnetic resonance imaging (MRI) session (1x fentanyl patches, 1x tramadol). We also excluded users of psychotropic medication, psychotic patients and patients with an acute major depression or bipolar disorder. Sixteen FM reported previous major depressive episodes, four of whom also had a history of generalized anxiety disorder and/or PTSD. None of the HC reported current or past psychopathological symptoms. We expected no relevant bias due to a highly standardized and randomized computer-controlled experimental test procedure. For a summary of the utilized diagnostic and clinical tools, see Table S1.

The study was approved by the ethics review board of the Medical Faculty, Ruhr University Bochum (15–5489). All participants gave written informed consent prior to participating in the study.

### 2.2 General design

The experiment was announced as an investigation of pain perception in FM. Participants were told that they would receive painful thermal stimuli (see section ‘Heat pain stimuli and threshold determination’) that would either be stopped by them or by a computer, and that they would afterwards have to rate these stimuli regarding their perceived intensity and unpleasantness (see section ‘Rating procedure’). The experiment followed a mixed between-within repeated measures design with “controllability” (2 levels: “self-controlled heat” and “computer-controlled heat”) as a within-subjects factor and “group” (2 levels: “FM” and “HC”) as a between-subjects factor. The entire test appointment lasted two to three hours, of which one hour was conducted in the MR. The investigation took place at the Department of Neurology, Berufsgenossenschaftliches Universitätsklinikum Bergmannsheil in Bochum between August 2019 and December 2020.

### 2.3 Experimental task

**Fig. 1:**
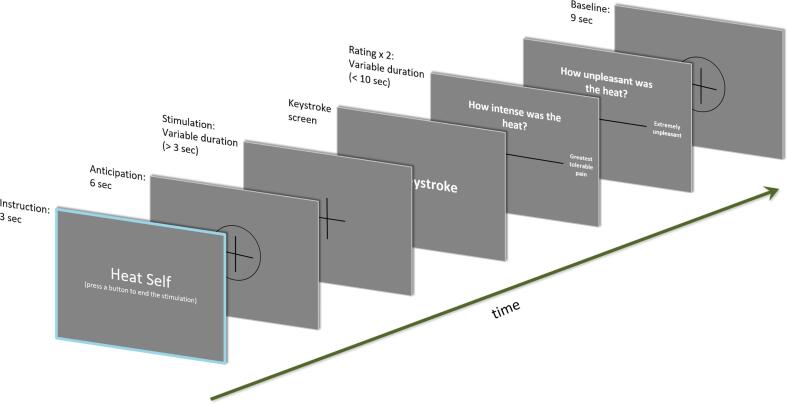
Sequence of an exemplary test trial.

The experiment consisted of four consecutive test blocks. Each block contained four repetitions of each condition (self-controlled or computer-controlled), making it 8 trials per block and 32 trials throughout the experiment. Before each trial (see Fig. 1), subjects were presented with a cue indicating the upcoming condition, followed by a 6-second anticipation phase. The next screen showed a crosshair, accompanied by the individual stimulus presentation. In self-controlled trials, participants ended the stimulation with a key stroke. In computer-controlled trials, the computer ended the stimulation. The duration of each computer-controlled trial was based on the mean of the previous two self-controlled runs. In this way, we obtained comparable stimulus presentation lengths for self- and computer-controlled trials. Due to this procedure, the first session of the experiment needed to start with two self-controlled rounds in order to have a starting point for the subsequent computer-controlled trials. We set the stimulus durations to a minimum of 3 s, regardless of possible keystrokes at an earlier point. Stimulation times (see Table 1) were not significantly different between groups for self-controlled (*t*(40.92) = -0.15, *p* =.88) or computer-controlled trials (*t*(41.92) = -0.34, *p* =.74).

**Table 1:** Stimulation thresholds, ratings and durations.

	*HC*	*FM*
Pain threshold (M (SD))	45.99 (2.3)	44.76 (2.41)
Pain tolerance (M (SD))	48.47 (2)	47.43 (2.04)
Calculated stimulus intensity (M (SD))	47.05 (2.07)	46.07 (2.1)
Adjusted stimulus intensity (M (SD))	47.52 (1.77)	45.17 (2.05)
		
Self-controlled pain (M (SD))	65.54 (14.12)	69.46 (13.16)
Computer-controlled pain (M (SD))	64.87 (14.74)	68.45 (13.27)
		
Self-controlled heat durations (M(SD))	6.91 (2.4)	7.02 (2.24)
Computer-controlled heat durations (M(SD))	6.95 (2.31)	7.19 (2.43)

*Note*. M and SD are used to represent mean and standard deviation. Pain thresholds and tolerance levels were assessed in order to calculate a preliminary stimulus intensity. Afterwards, this calculated stimulus intensity was adjusted using a MATLAB routine. Thresholds as well as calculated and adjusted stimulus intensities are listed in degrees Celsius (°C). Perceived stimulus intensities were rated on a scale from 0 to 100. Heat durations are listed in seconds (s).

When self-controlled trials were terminated, a screen displaying the word ’keystroke’ was shown. To control for motor responses, computer-controlled trials were followed by the same keystroke screen, in this case indicating that pressing a button would take the participants to the next screen. Following the keystroke, subjects were instructed to evaluate the stimulus intensity and unpleasantness on visual analog scales (VAS, see section ‘Rating procedure’) using a LUMItouch button box (Photon Control, Inc, Burnaby, BC, Canada). On the final screen of each trial, subjects were presented with another circled cross for 9 s (baseline condition). Prior to the actual fMRI experiment, subjects were familiarized with the instructions displayed during the experiment and with the rating procedure. In order to verify whether participants did actually feel in control during self-controlled trials, they were asked to rate the perceived control over self-controlled and computer-controlled stimuli following the experiment on a scale from 0 ‘no control’ to 10 ‘complete control’. The audiovisual Visuastim Digital Box (Resonance Technology Inc., USA) was used for the visual presentation of instructions and scales.

Prior to each trial, participants were informed whether the following round would be terminated by the computer (computer-controlled) or by the subjects themselves (self-controlled). A short anticipation phase of 6 s was then followed by the variable stimulation interval (with a minimum of 3 s) and a screen showing the term ’keystroke’, indicating a preceded (self-controlled trials) or required (computer-controlled trials) keystroke. Subsequently, the subjective stimulus intensity and unpleasantness were rated via visual analogue scales. After the rating procedure, the last 9 s of each trial were used as a baseline period.

### 2.4 Heat pain stimuli and threshold determination

Experimental heat pain was induced using a 3x3cm contact thermode (PATHWAY Pain & Sensory Evaluation System, Medoc ltd. Advanced Medical System, Israel) on the participants’ left thenar. During the experiment, the device was located in the control room and bridged to the scanner. Individual heat pain threshold and tolerance were determined using MEDOC Main Station 6.3. Ascending thermal stimulation cycles were presented and the mean value of the last three out of five consecutive threshold ratings was calculated. For pain threshold, subjects were asked to stop the stimulation via a mouse button when they started perceiving the stimulus as just painful, for pain tolerance they stopped at the maximum bearable heat. We calculated the specific stimulation intensity by building the mean between pain threshold and pain tolerance and adjusting the resulting intensity by running a Matlab routine. In a series of consecutive adjustment steps, a tonic heat stimulus of 27 s (ramp: 0.15 s per °C) was presented, followed by a VAS (see section ‘Rating procedure’) for intensity and unpleasantness ratings. Heat levels of the subsequent trials were adapted based on the respective intensity ratings until two consecutive ratings were in a range between 60 and 70. The adjusted stimulation intensity was used for both, the self-controlled and the computer controlled condition.

### 2.5 Rating procedure

During the examination, participants were asked to rate perceived intensity and unpleasantness of the presented heat pain stimuli after each trial on a VAS with the anchors 0 ’not painful’ to 100 ’greatest tolerable pain’, and 0 ’not unpleasant’ to 100 ’extremely unpleasant’.

### 2.6 MRI data acquisition

Anatomical and functional MRI data were obtained on a Philips Achieva 3 T MRI scanner using a 32-channel standard head coil, packed with foam pads for fixation purposes. Functional scans were conducted using an echo planar (*EPI*) T2* sensitive sequence with 48 axial slices per volume and an isotropic voxel resolution of 2.3 mm (slice thickness 2.3 mm, TR = 2.5 s, TE = 22 ms, flip angle 82°, FOV 220x133.9 mm^2^). Each functional imaging sequence was initiated by three dummy scans that were not taken into account during later evaluation steps. A high-resolution Magnetization Prepared Rapid Gradient (MPRAGE), comprising 204 sagittal slices, was obtained for each subject (slice thickness 1 mm, TR = 7 s, TE = 3.2 s, flip angle 8°, 1x1x1mm voxel size, FOV 256x204mm^2^).

### 2.7 fMRI data analysis

Functional MRI data were evaluated with SPM12 (Wellcome Trust Centre for Neuroimaging, London, UK) in Matlab R2020b (Mathworks Inc., Natick, MA). All images were realigned to the fourth volume, slice time corrected, co-registered to the individual anatomical scan (MPRAGE), spatially normalized to the MNI template (Montreal Neurological Institute) (Evans et al., 1993) and smoothed with a 4 mm Gaussian kernel (full-width at half-maximum).

Contrast images were calculated for self-controlled and computer-controlled heat, comparing each stimulus condition to the 9 s baseline conditions of all trials. Subsequently, comparisons between self-controlled and computer-controlled heat were calculated for each subject. We applied a family-wise error (FWE) correction for multiple comparisons at a threshold of *p* <.05. In consideration of the relatively small sample size and previous studies showing neural activations of specific small brain areas, we decided to perform further analyses using a less conservative global threshold of *p*_(uncor.)_ < 0.001 that has also been used in the preceding studies by Wiech et al. (2006 & 2014). For group contrasts the significance level was set to *p*_(uncor.)_ ≤ 0.005. Search volumes were defined by centering sphere ROIs around previously established coordinates (right VLPFC: 36, 45, 15 (8 mm), left VLPFC: −36, 45, 15 (8 mm) (VLPFC peak voxels during behavioral control (Wiech et al., 2014)); right DLPFC: 33, 33, 39 (15 mm), left DLPFC: –33, 33, 39 (15 mm); right OFC: 24, 27, −15 (15 mm), left OFC: −24. 27, −15 (15 mm) (peak voxels of Wiech et al. (2006)). Keuken and Forstmann’s 7 T Atlas (Keuken and Forstmann, 2015) was utilized for the periaqueductal gray (PAG). Other relevant brain areas such as cerebellum, insular, thalamic, primary and secondary somatosensory cortices (SI, SII) were defined based on the automated anatomical labeling (AAL) atlas (Tzourio-Mazoyer et al., 2002).

### 2.8 Functional connectivity analysis

FC fMRI analyses were performed in MATLAB R2020b using the CONN functional connectivity toolbox (Whitfield-Gabrieli and Nieto-Castanon, 2012). For each participant, preprocessed functional and structural MRI data, derived from the previous evaluation steps, was first corrected for physiological confounding signals by means of the CompCor approach (Behzadi et al., 2007). Principal components of white matter (WM) and cerebrospinal fluid (CSF) were identified by segmentation of the anatomical MPRAGE images and later entered as confound variables in the denoising step of the FC analysis routine. This procedure facilitates the reduction of spatial correlations resulting from physiological noise. In addition, our denoising pipeline included a confound variable for realignment as well as for scrubbing of head motion-induced artifacts. Subsequently, BOLD-timeseries were extracted and band-pass-filtered (0.008–0.09 Hz) to reduce the influence of noise.

Seed-based data analysis was performed by computing the temporal correlation between the BOLD signals from a given voxel to all other voxels in the brain. Within the framework of the seed-to-voxel FC analysis we conducted, pain inhibition and reappraisal areas were used as seed ROIs (VLPFC, DLPFC, dACC, PAG) to examine group differences regarding FC during self-controlled heat trials. The significance level for these group comparisons was set to *p*_(uncor.)_ ≤ 0.001. The aim of this approach was to specify FM-related deficiencies in terms of neural pain modulation / inhibition regarding FC of relevant brain regions. To further specify the exact processes potentially disrupted in FM, we defined bilateral activation clusters in the AI based on activations from the main contrast of pain (heat > pain) and used these clusters as ROIs to calculate another seed-to-voxel FC analysis. In this manner, we wanted to explore a possible decoupling of pain-processing (AI) and prefrontal areas (VLPFC, DLPFC) and thus identify the precise processes disrupted in FM.

### 2.9 Anatomical data analysis

Group differences in local concentrations of brain tissue were assessed across the entire brain through voxel-based morphometry (VBM) which was implemented in SPM12 using the Computational Anatomy Toolbox CAT12 (Structural Brain Mapping Group, Jena, Germany). High-resolution structural MPRAGE images were segmented, normalized to MNI standard space and smoothed with an isotropic Gaussian kernel of 8 mm. Total intracranial volume (TIV) was estimated as the sum of the three main brain tissue volumes. In order to compare GM volumes of FM and HC, a two-sample *t* test was conducted in SPM12. TIV was used as a covariate during the analysis in order to correct for differences in brain size. Focusing on the predefined (bilateral) ROIs from our prior analyses (VLPFC, DLPFC, OFC, dACC), *t* tests were calculated to compare group-wise GM volumes in these regions. The significance level was set to *p* <.05.

## 3 Results

**Fig. 2:**
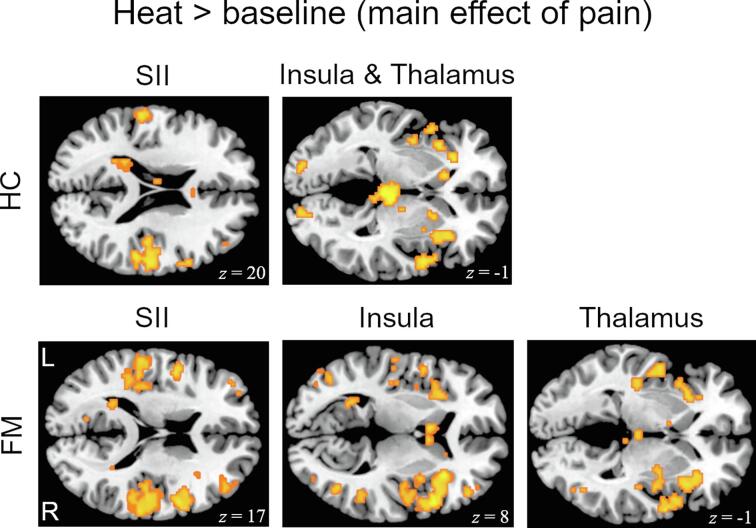
Group-specific brain responses to heat pain compared to baseline (main effect of pain). Significant activity during self- and computer-controlled heat stimulation combined, compared to baseline intervals was found, inter alia, within the illustrated clusters of insula, SII and thalamus (thresholded at *p*_(uncorr.)_ < 0.001 for display purposes). MNI coordinates are depicted.

For the main effect of pain (self-controlled and computer-controlled heat pain trials combined compared to baseline), HC and FM showed bilateral activations in pain-related sensory, limbic and associative brain regions, such as the insula, SII (*p* <.05, FWE corrected), thalamus, anterior cingulate cortex (ACC) and caudate nucleus (*p*_(uncorr.)_ < 0.001) (see Fig. 2). However, FM displayed significantly less activation compared to HC in the medial frontal gyrus (MFG) and bilateral thalamus.

### 3.1 FM display disturbed activation of the VLPFC and DLPFC during self-controlled heat stimulation

**Fig. 3:**
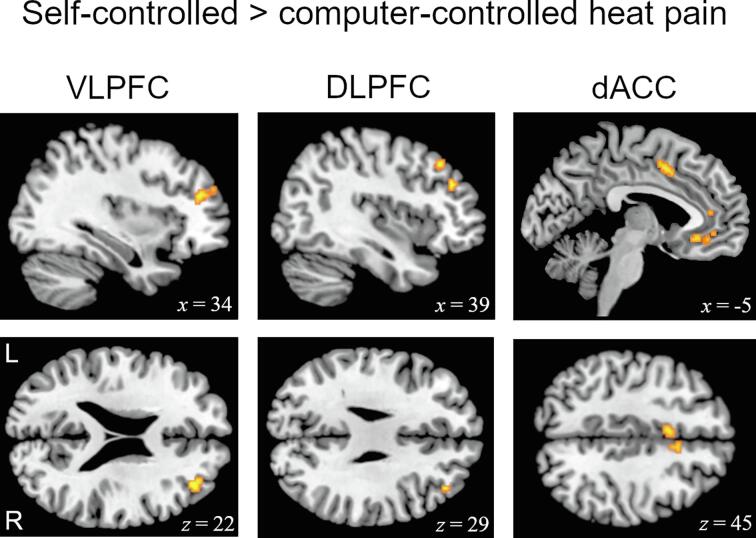
Brain responses to self-controlled compared to computer-controlled heat pain in HC. Significantly greater activity during self-controlled heat stimulation compared to computer-controlled trials was found in HC within the illustrated clusters of right VLPFC (34, 42, 22) (*p*(FWE) < 0.05), right DLPFC (39, 37, 29) and bilateral dACC (9, 10, 40 and −5, 8, 45) (*p*_(uncorr.)_ < 0.001). MNI coordinates are depicted.

Comparing neural activity during self-controlled versus computer-controlled heat pain stimulation in the HC group, we observed a significant activation cluster within right VLPFC (*p* <.05, FWE corrected). Using a less conservative threshold (*p*_(uncorr.)_ < 0.001), we found additional activations in left VLPFC, right DLPFC and bilateral dACC (see Fig. 3 and Table S2). Apart from this, the contrast revealed significant activations on a whole-brain level in a range of other structures, such as bilateral SMA, bilateral insula, bilateral rolandic operculum (SII), bilateral caudate nucleus and left supramarginal gyrus.

In contrast, FM showed no significant activation within the relevant structures of the prefrontal cortex (PFC) for self-controlled compared to computer-controlled trials. Significant differences were only observed in clusters of the right midcingulate cortex (MCC; 9, 17, 38; *z* = 3.55) and left precentral cortex (-35, -27, 56; *z* = 3.44).

Group contrasts testing for greater activation in HC compared to FM affirmatively revealed significant clusters of activation in right VLPFC and right DLPFC (see Table S3). Additionally, we found distinct group differences, inter alia, in right putamen, right hypothalamus, right caudate nucleus, left amygdala and left inferior frontal gyrus (IFG).

### 3.2 FM activate the limbic system, instead of the OFC, during computer-controlled heat

Testing the opposite contrast (computer-controlled > self-controlled heat) in HC, we found significant activation clusters in the right OFC (25, 44, -8; *z* = 2.95; *p* =.001), orbital MFG (0, 60, -8; *z* = 3.49; *p* <.001), right hippocampus (21, -20, -17; *z* = 3.69; *p* <.001) and left parahippocampal gyrus (-16, -25, –22; *z* = 2.84; *p* =.001).

**Fig. 4:**
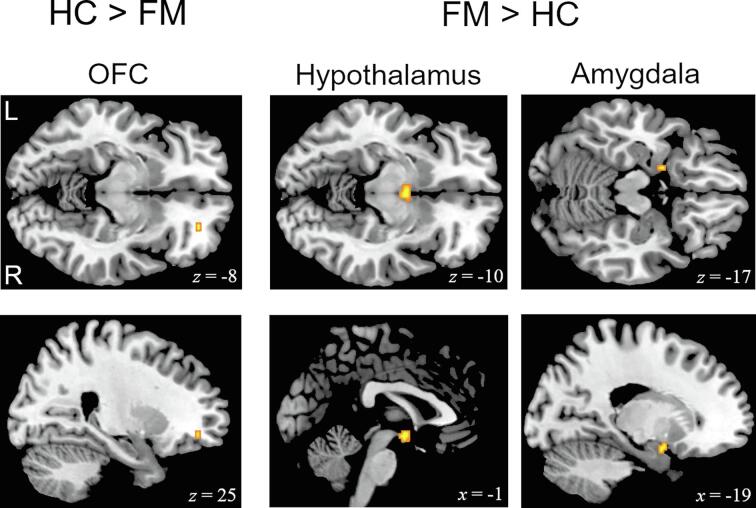
Brain regions showing significantly different levels of activation in healthy controls (HC) and patients with fibromyalgia (FM) for the contrast “computer-controlled > self-controlled heat pain”. Relevant clusters are located in the right orbitofrontal cortex (OFC) (25, 44–8), Hypothalamus (-1, −4, −10) and left Amygdala (-19, 1, −17).

Despite relatively similar activation patterns in FM, patients displayed no significant activation of the OFC (significant group difference HC > FM; 25, 44, −8; *z* = 2.68; *p* =.004, see Fig. 4). For FM, we detected significantly greater activity compared to HC in the left amygdala (-19, 1, -17; *z* = 2.81; *p* =.002), hypothalamus (0, -4, -10; *z* = 3.06; *p* =.001), right putamen (25, 1, 1; *z* = 3.27; *p* =.001) and right caudate nucleus (16, 19, 8; *z* = 2.95; *p* =.002) (significant group difference FM > HC, see Fig. 4).

### 3.3 Functional connectivity between (prefrontal) pain modulatory reappraisal areas and the remaining brain is decreased in FM

**Fig. 5:**
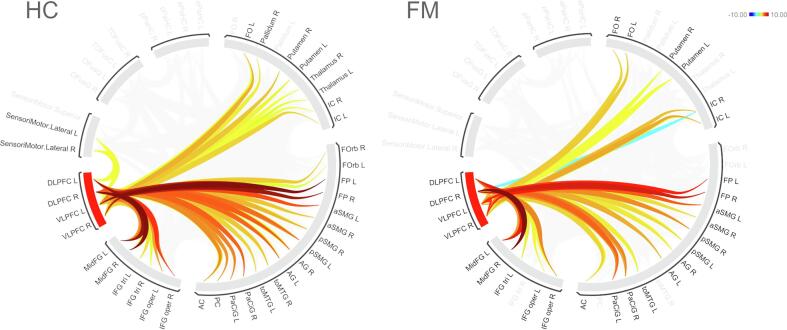
Cumulated group-wise Functional Connectivity (FC) of bilateral VLPFC and DLPFC during self-controlled heat stimulation. Group-wise FC of each ROI is illustrated through lines that are colored proportionally to the F statistics (color legend in the top right corner). R: right; L: left; VLPFC: ventrolateral prefrontal cortex; DLPFC: dorsolateral prefrontal cortex; AG: angular gyrus; IC: insular cortex; FP: frontal pole; FOrb: frontal orbital cortex; MidFG: middle frontal gyrus; PaCiG: paracingulate gyrus; OFusG: occipital fusiform gyrus; TOFusC: temporal occipital fusiform cortex; toMTG: temporooccipital middle temporal gyrus; IFG oper: inferior frontal gyrus, pars opercularis; IFG tri: inferior frontal gyrus, pars triangularis; pSMG: posterior supramarginal gyrus; aSMG: anterior supramarginal gyrus; aPaHC: anterior parahippocampal gyrus; pPaHC: posterior parahippocampal gyrus; PC: posterior cingulate cortex; AC: anterior cingulate cortex; connection threshold of *p*(FDR) < 0.05 used for the illustration.

Using the right VLPFC ROI as a seed, FM showed decreased functional coupling with a number of brain regions compared to HC, such as the right parahippocampal gyrus, bilateral OFC, right angular gyrus, left supramarginal gyrus and posterior cingulate cortex (PCC) (see Fig. 5). As for the left VLPFC seed, FM displayed decreased connectivity compared to HC, inter alia, with right postcentral and supramarginal gyri, bilateral thalamus, PCC, ACC, left inferior frontal gyrus (IFG), right caudate nucleus and right insula. Examining the right DLPFC, FM showed decreased coupling with right supramarginal gyrus, right precentral and bilateral primary motor cortices (M1), right insula, PAG, right precuneus and right thalamus. A similar pattern was detected for the dACC, which showed decreased connectivity with PAG, left angular gyrus, right insula and left SMA in FM. In contrast, FM showed almost no relevant increases in FC within the main seed ROIs investigated in our study compared to HC.

Using the anterior insula (AI) ROIs, both HC and FM displayed significant connectivity of the insular clusters and bilateral VLPFC, but not with bilateral DLPFC. Group contrasts revealed no significant differences.

### 3.4 Gray matter volumes of relevant pain modulatory areas are decreased in FM

**Fig. 6:**
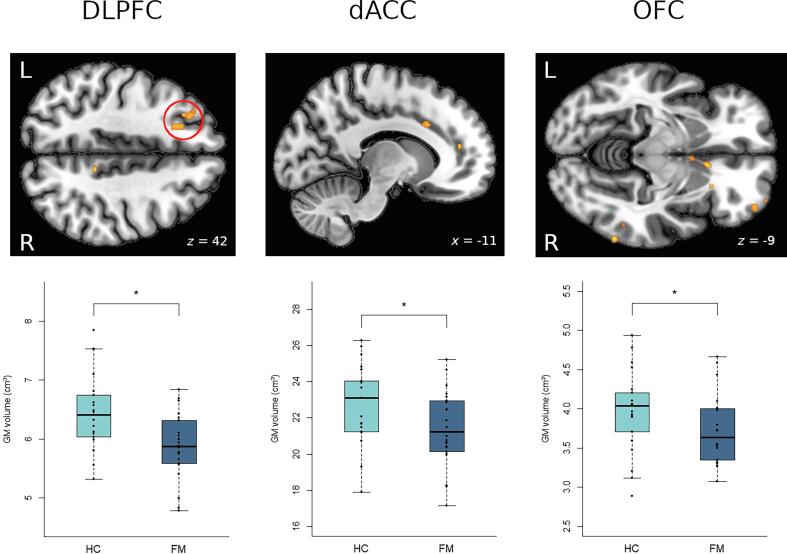
Group comparisons of gray matter (GM) volume in our pre-defined regions of interest (ROIs). Brain gray matter (GM) volume decreases in FM patients compared to HC in the right dorsolateral prefrontal cortex (DLPFC), dorsal anterior cingulate cortex (dACC) and right orbitofrontal cortex (OFC); p ≤ 0.001; B). Bar charts of the mean GM volumes (cm^3^) for HC and FM in the significant clusters. Individual GM mean values for each participant are indicated by black dots. * *p* <.05. L: left; R: right; HC: healthy controls; FM: patients with fibromyalgia.

Focusing on the predefined ROIs from our hypotheses, *t* tests revealed that FM patients had decreased GM volumes in the left DLPFC (*t*(34.62) = 2.37, *p* =.001), dACC (*t*(40.88) = 2.09, *p* =.02) and right OFC (*t*(38.82) = 1.69, *p* =.05, see Fig. 6), whereas volumes of bilateral VLPFC and right DLPFC did not differ significantly between groups.

## 4 Discussion

In contrast to the obsolescent concept of pain as a direct readout of nociceptive input, contemporary research acknowledges the complex nature of pain perception and its sensitivity to cognitive manipulations. The primary aim of the present study was to explore the neural modulation of experimental pain induced through experienced control in FM and to uncover possible condition-specific changes compared to HC. Our data provide clear indications for disrupted neural pain modulatory processes in FM, mainly affecting frontal components of the pain inhibitory network (VLPFC, DLPFC, OFC). While activation patterns found in HC strongly resembled previous findings (Salomons et al., 2004, Wiech et al., 2006), FM displayed significantly decreased activity, decreased FC as well as decreased GM volume in relevant pain modulatory brain regions involving VLPFC, DLPFC, OFC and dACC.

### 4.1 fMRI results

HC and FM displayed similar activations of the pain network during heat pain compared to baseline. However, patients showed less activation in MFG and bilateral thalamus, which has been shown before (Burgmer et al., 2009b, Gracely et al., 2002). Decreased thalamic activity may be interpreted as the consequence of tonic inhibition from persistent afferent pain input in FM (Gracely et al., 2002).

In line with previous investigations, HC displayed increased activity in right VLPFC as well as right DLPFC and bilateral dACC during self-controlled compared to computer-controlled trials (Salomons et al., 2007, Salomons et al., 2004, Wiech et al., 2014, 2006). These brain regions have been proven to play a crucial role in voluntary reappraisal (Kalisch et al., 2005) and descending pain modulatory processes (Tracey and Mantyh, 2007). The ability to control painful stimuli is thought to cause a reassessment (reappraisal) of the aversive situation, eventually making pain less threatening and painful (Leeuw et al., 2007). With regard to such neural reappraisal processes, the VLPFC has been shown to be of particular importance (Kalisch et al., 2006, Mohr et al., 2012, Salomons et al., 2007, Wiech et al., 2006). For instance, Wiech et al. (2006) found subjects with a strong internal locus of control to activate the area less when they had no control over pain. Similar activations have been demonstrated during other types of reappraisal (Lévesque et al., 2004, Ochsner et al., 2004, Phan et al., 2005, Wager et al., 2008). Interestingly, we found no VLPFC activity in FM. This may serve as an indication for dysfunctional brain circuitries in the domain of control-induced neural pain modulation, suggesting that FM might be severely limited in their cognitive ability to efficiently cope with acute pain.

Another prefrontal area critically involved in pain modulation is the DLPFC, which is ascribed a pivotal role in the regulation and maintenance of top-down modulation and control of behavioral responses (Brosnan and Wiegand, 2017, Edin et al., 2009, Miller and Cohen, 2001). For example, DLPFC activity is negatively correlated with pain catastrophizing scores, a measure of uncontrollability (Seminowicz and Davis, 2006), during intense pain. Beyond that, the DLPFC is also seen as a key node of pain inhibitory networks. For instance, participants demonstrated increased activation of bilateral DLPFC when given instructions to suppress experimental heat pain (Freund et al., 2009) and activity of the area is associated with a reduction of perceived pain intensity and unpleasantness (Lorenz et al., 2003, Raij et al., 2009). Furthermore, it has been discovered that transient lesioning of the DLPFC using transcranial magnetic stimulation (TMS) abolishes placebo analgesia (Krummenacher et al., 2010).

A possible explanation for the observed alterations in FM during self-controlled trials might be a more pronounced external locus of control which has repeatedly been demonstrated in chronic pain patients (Gustafsson and Gaston-Johansson, 1996, Shuster et al., 2009) and is negatively associated with VLPFC activity (Wiech et al., 2006). This is supported by significantly higher values on the external “helplessness” and “fatalism” scales (Krampen, 1981) (see Table S1) in FM compared to HC. It is conceivable that the externalized locus of control as such hindered FM from truly feeling in control in our experimental setting. This response might arguably be similar to their response towards chronic pain in daily life.

Cognitive and emotional variables can decisively affect the individual experience of pain. However, a positive compared to a negative emotional context can reduce the perceived pain intensity in HC but not in FM (Kamping et al., 2013). Perceived control over noxious stimuli modulates pain perception similarly to a positive emotional context, whereas uncontrollable pain is comparable to a negative emotional context. In this way, pain modulation through emotional context and perceived control show clear similarities in FM, suggesting widespread dysfunctions of the pain modulating system that affect various modulatory processes.

Testing for greater activity during computer-controlled compared to self-controlled pain in HC revealed significant activation in the OFC (Wiech et al., 2006). Such activity can typically be observed during painful (Wiech et al., 2005) and affective states in general (O’Doherty, 2004). In comparison, FM displayed no verifiable activation of the OFC. This demonstrates another dysfunctional subdomain of neural pain processing in FM, as the OFC plays a crucial role in the mediation of pain inhibition (Becker et al., 2017). Instead, the evaluation of our patient data revealed enhanced activity of structures within the limbic system that are typically associated with emotional responses and affective states (amygdala, parahippocampal gyrus) (Davis and Whalen, 2001, Gosselin, 2006, Phelps and LeDoux, 2005). Comparable activations during experimental pain have previously been demonstrated for low perceived levels of control as well as high levels of pain-related fear and pain catastrophizing (amygdala and hippocampus) (Gracely, 2004, Hsiao et al., 2020, Lu et al., 2010). In chronic pain disorders, pain-related activation of the amygdala has primarily been ascribed a pain-enhancing influence (Palazzo et al., 2008). Considering these findings, the elevated amygdala activity in our FM sample corresponds to previous investigations on sensory and affective aspects of pain processing in chronic pain (Giesecke et al., 2005, Jensen et al., 2009).

### 4.2 Functional connectivity

Our FC results are largely in line with a previous investigation on resting-state data by Flodin et al (2014) who reported decreased connectivity between pain-related regions and the remaining brain. According to Flodin et al. (2014), FM are characterized by a weaker coupling between pain areas and prefrontal / sensorimotor areas. This might indicate a less efficient system level control of pain circuits, pointing towards deficiencies in pain modulation. An earlier investigation on FM by Jensen et al. (2012) found decreased FC of pain inhibitory networks, primarily involving the rostral ACC (rACC) and thalamus. Our FC findings represent an extension of these results, providing an exploration of further pain modulatory brain areas and demonstrating specific effects related to control-induced analgesia. Another study found bilateral DLPFC to suppress activity in both the thalamus and right insula, thus reducing pain sensitization during uncontrollable pain (Bräscher et al., 2016). The observed FC decreases between DLPFC and key pain-processing areas such as thalamus and insula during self-controlled trials paint a more accurate picture of the descending pain-inhibiting pathways that appear to be disrupted in FM. FM showed no functional decoupling of AI and VLPFC, suggesting that the observed pain modulatory dysfunctions of the VLPFC in FM are most probably affiliated to regionally disrupted processing rather than reduced FC with pain-processing regions as AI.

### 4.3 Gray matter changes

Group comparisons demonstrated striking patterns of morphometric changes in FM, affecting brain regions that are related to pain modulation. These findings are largely in line with previous investigations on brain structural changes in FM that have documented distinct GM decreases in relevant pain modulatory structures of cingulate, prefrontal and orbitofrontal cortices (Burgmer et al., 2009a, Ceko et al., 2013, Jensen et al., 2013, Kuchinad et al., 2007, Robinson et al., 2011). In view of the reported findings and our hypotheses, the GM decreases we found in DLPFC and dACC are of particular interest. Both structures have previously been proven to be decreased in FM (Burgmer et al., 2009a, Lutz et al., 2008). Beyond that, decreased DLPFC volume has been observed in a variety of chronic pain populations (Erpelding et al., 2016, Rodriguez-Raecke et al., 2013, Valfrè et al., 2007).

The FM-related functional changes reported above are largely reflected in our brain-morphometric findings. Taking account of our functional and structural results, the prefrontal cortex is at the center of the dysfunctional changes. Apart from the considerable functional abnormalities of the VLPFC in FM, the DLPFC demonstrated comparable functional as well as distinct structural changes. With reference to the vital importance of the DLPFC for a large number of complex cognitive processes, the findings described above are particularly meaningful. Thus, our findings provide evidence for far-reaching limitations concerning pain-modulation in FM and might offer an insight into the extent to which dysfunctional neural processes contribute to the maintenance of chronic pain on a daily basis.

Limitations

Some methodological characteristics of this study should be considered when interpreting the results. While we made sure that all of our participants had a secure clinical FM diagnosis, there was some heterogeneity regarding disease duration in our sample. This variance was not considered in our analysis and could theoretically have an effect on the results. In addition, our study design and the relatively small sample size do not allow clear conclusions as to whether (part of) the measured changes may have been time-related. Subsequent investigations could focus more thoroughly on potential correlations of the above-mentioned critical brain areas with behavioral / psychological measures (i.e., pain catastrophizing, coping behavior, locus of control).

### 4.4 Conclusions

FM displayed broad functional and structural changes of pain modulatory neural networks, especially involving prefrontal, cingulate and limbic brain areas. The VLPFC and DLPFC in particular appear to be at the center of these neural changes. Considering the pivotal role of the DLPFC for pain modulatory processes, the substantial negative impact of the altered pain processing brain circuitries established in our examination through a variety of functional and structural analytical methods becomes apparent. Identifying the neural network that is involved in pain inhibitory processes due to controllability could well be targeted within the framework of clinical pain therapy (e.g., TMS, neurofeedback, cognitive behavioral trainings). A number of such therapeutic approaches have already shown long-lasting analgesic effects in FM, including repetitive TMS to the right primary motor cortex (Passard et al., 2007) and neurofeedback trainings of sensorimotor rhythm and alpha brain waves (Wu et al., 2021). In this respect, the regional functional and structural changes we have detected could be used for exploratory planning of new treatment concepts.

## Declaration of Competing Interest

The authors declare that they have no known competing financial interests or personal relationships that could have appeared to influence the work reported in this paper.

## Data Availability

Data will be made available on request.

## References

[b0005] Becker S., Gandhi W., Pomares F., Wager T.D., Schweinhardt P. (2017). Orbitofrontal cortex mediates pain inhibition by monetary reward. Soc. Cogn. Affect. Neurosci..

[b0010] Behzadi Y., Restom K., Liau J., Liu T.T. (2007). A component based noise correction method (CompCor) for BOLD and perfusion based fMRI. NeuroImage.

[b0015] Borckardt J.J., Reeves S.T., Frohman H., Madan A., Jensen M.P., Patterson D., Barth K., Smith R.A., Gracely R., George M.S. (2011). Fast left prefrontal rTMS acutely suppresses analgesic effects of perceived controllability on the emotional component of pain experience. Pain.

[b0020] Bräscher A.-K., Becker S., Hoeppli M.-E., Schweinhardt P. (2016). Different Brain Circuitries Mediating Controllable and Uncontrollable Pain. J. Neurosci..

[b0025] Brosnan M.B., Wiegand I. (2017). The Dorsolateral Prefrontal Cortex, a Dynamic Cortical Area to Enhance Top-Down Attentional Control. J. Neurosci..

[b0030] Burgmer M., Gaubitz M., Konrad C., Wrenger M., Hilgart S., Heuft G., Pfleiderer B. (2009). Decreased Gray Matter Volumes in the Cingulo-Frontal Cortex and the Amygdala in Patients With Fibromyalgia. Psychosom. Med..

[b0035] Burgmer M., Pogatzkizahn E., Gaubitz M., Wessoleck E., Heuft G., Pfleiderer B. (2009). Altered brain activity during pain processing in fibromyalgia. NeuroImage.

[b0040] Ceko M., Bushnell M.C., Fitzcharles M.-A., Schweinhardt P. (2013). Fibromyalgia interacts with age to change the brain. NeuroImage Clin..

[b0045] Davis M., Whalen P.J. (2001). The amygdala: vigilance and emotion. Mol. Psychiatry.

[b0050] Edin F., Klingberg T., Johansson P., McNab F., Tegnér J., Compte A. (2009). Mechanism for top-down control of working memory capacity. Proc. Natl. Acad. Sci..

[b0055] Erpelding N., Simons L., Lebel A., Serrano P., Pielech M., Prabhu S., Becerra L., Borsook D. (2016). Rapid treatment-induced brain changes in pediatric CRPS. Brain Struct. Funct..

[b0060] Evans, A.C., Collins, D.L., Mills, S.R., Brown, E.D., Kelly, R.L., Peters, T.M., 1993. 3D statistical neuroanatomical models from 305 MRI volumes, in: 1993 IEEE Conference Record Nuclear Science Symposium and Medical Imaging Conference. Presented at the 1993 IEEE Conference Record Nuclear Science Symposium and Medical Imaging Conference, IEEE, San Francisco, CA, USA, pp. 1813–1817. https://doi.org/10.1109/NSSMIC.1993.373602.

[b0065] Flodin P., Martinsen S., Löfgren M., Bileviciute-Ljungar I., Kosek E., Fransson P. (2014). Fibromyalgia is associated with decreased connectivity between pain-and sensorimotor brain areas. Brain Connect..

[b0070] Freund W., Klug R., Weber F., Stuber G., Schmitz B., Wunderlich A.P. (2009). Perception and suppression of thermally induced pain: A fMRI study. Somatosens. Mot. Res..

[b0075] Giesecke T., Gracely R.H., Williams D.A., Geisser M.E., Petzke F.W., Clauw D.J. (2005). The relationship between depression, clinical pain, and experimental pain in a chronic pain cohort. Arthritis Rheum..

[b0080] Gosselin N. (2006). Emotional responses to unpleasant music correlates with damage to the parahippocampal cortex. Brain.

[b0085] Gracely R.H. (2004). Pain catastrophizing and neural responses to pain among persons with fibromyalgia. Brain.

[b0090] Gracely R., Petzke F., Wolf J.M., Clauw D.J. (2002). Functional magnetic resonance imaging evidence of augmented pain processing in fibromyalgia. Arthritis Rheum..

[b0095] Gustafsson M., Gaston-Johansson F. (1996). Pain intensity and health locus of control: a comparison of patients with fibromyalgia syndrome and rheumatoid arthritis. Patient Educ. Couns..

[b0100] Hsiao F.-J., Chen W.-T., Ko Y.-C., Liu H.-Y., Wang Y.-F., Chen S.-P., Lai K.-L., Lin H.-Y., Coppola G., Wang S.-J. (2020). Neuromagnetic Amygdala Response to Pain-Related Fear as a Brain Signature of Fibromyalgia. Pain Ther..

[b0105] Jensen, K.B., Loitoile, R., Kosek, E., Petzke, F., Carville, S., Fransson, P., Marcus, H., Williams, S.C., Choy, E., Mainguy, Y., Vitton, O., Gracely, R.H., Gollub, R., Ingvar, M., Kong, J., 2012. Patients with Fibromyalgia Display Less Functional Connectivity in the Brain’s Pain Inhibitory Network. Mol. Pain 8, 1744-8069-8–32. https://doi.org/10.1186/1744-8069-8-32. 10.1186/1744-8069-8-32 PMC3404927 22537768

[b0110] Jensen K.B., Kosek E., Petzke F., Carville S., Fransson P., Marcus H., Williams S.C.R., Choy E., Giesecke T., Mainguy Y., Gracely R., Ingvar M. (2009). Evidence of dysfunctional pain inhibition in Fibromyalgia reflected in rACC during provoked pain. Pain.

[b0115] Jensen K.B., Srinivasan P., Spaeth R., Tan Y., Kosek E., Petzke F., Carville S., Fransson P., Marcus H., Williams S.C.R., Choy E., Vitton O., Gracely R., Ingvar M., Kong J. (2013). Overlapping Structural and Functional Brain Changes in Patients With Long-Term Exposure to Fibromyalgia Pain: Brain Changes in Long-Term Fibromyalgia. Arthritis Rheum..

[b0120] Kalisch R., Wiech K., Critchley H.D., Seymour B., O’Doherty J.P., Oakley D.A., Allen P., Dolan R.J. (2005). Anxiety Reduction through Detachment: Subjective, Physiological, and Neural Effects. J. Cogn. Neurosci..

[b0125] Kalisch R., Wiech K., Herrmann K., Dolan R.J. (2006). Neural correlates of self-distraction from anxiety and a process model of cognitive emotion regulation. J. Cogn. Neurosci..

[b0130] Kamping S., Bomba I.C., Kanske P., Diesch E., Flor H. (2013). Deficient modulation of pain by a positive emotional context in fibromyalgia patients. Pain.

[b0135] Keuken M.C., Forstmann B.U. (2015). A probabilistic atlas of the basal ganglia using 7 T MRI. Data Brief.

[b0140] Krampen, G., 1981. IPC-Fragebogen zu Kontrollüberzeugungen.

[b0145] Krummenacher P., Candia V., Folkers G., Schedlowski M., Schönbächler G. (2010). Prefrontal cortex modulates placebo analgesia. Pain.

[b0150] Kuchinad A., Schweinhardt P., Seminowicz D.A., Wood P.B., Chizh B.A., Bushnell M.C. (2007). Accelerated Brain Gray Matter Loss in Fibromyalgia Patients: Premature Aging of the Brain?. J. Neurosci..

[b0155] Leeuw M., Goossens M.E.J.B., Linton S.J., Crombez G., Boersma K., Vlaeyen J.W.S. (2007). The Fear-Avoidance Model of Musculoskeletal Pain: Current State of Scientific Evidence. J. Behav. Med..

[b0160] Lévesque J., Joanette Y., Mensour B., Beaudoin G., Leroux J.-M., Bourgouin P., Beauregard M. (2004). Neural basis of emotional self-regulation in childhood. Neuroscience.

[b0165] Lorenz J., Minoshima S., Casey K.L. (2003). Keeping pain out of mind: the role of the dorsolateral prefrontal cortex in pain modulation. Brain.

[b0170] Lu H.-C., Hsieh J.-C., Lu C.-L., Niddam D.M., Wu Y.-T., Yeh T.-C., Cheng C.-M., Chang F.-Y., Lee S.-D. (2010). Neuronal correlates in the modulation of placebo analgesia in experimentally-induced esophageal pain: A 3T-fMRI study. Pain.

[b0175] Lutz J., Jäger L., de Quervain D., Krauseneck T., Padberg F., Wichnalek M., Beyer A., Stahl R., Zirngibl B., Morhard D., Reiser M., Schelling G. (2008). White and gray matter abnormalities in the brain of patients with fibromyalgia: A diffusion-tensor and volumetric imaging study. Arthritis Rheum..

[b0180] Miller E.K., Cohen J.D. (2001). An Integrative Theory of Prefrontal Cortex Function. Annu. Rev. Neurosci..

[b0185] Mohr C., Leyendecker S., Petersen D., Helmchen C. (2012). Effects of perceived and exerted pain control on neural activity during pain relief in experimental heat hyperalgesia: A fMRI study. Eur. J. Pain.

[b0190] Müller M.J. (2011). Helplessness and perceived pain intensity: relations to cortisol concentrations after electrocutaneous stimulation in healthy young men. Biopsychosoc. Med..

[b0195] O’Doherty J.P. (2004). Reward representations and reward-related learning in the human brain: insights from neuroimaging. Curr. Opin. Neurobiol..

[b0200] Ochsner K.N., Ray R.D., Cooper J.C., Robertson E.R., Chopra S., Gabrieli J.D.E., Gross J.J. (2004). For better or for worse: neural systems supporting the cognitive down- and up-regulation of negative emotion. NeuroImage.

[b0205] Oldfield R.C. (1971). The assessment and analysis of handedness: The Edinburgh Inventory. Neuropsychologia.

[b0210] Palazzo E., Fu Y., Ji G., Maione S., Neugebauer V. (2008). Group III mGluR7 and mGluR8 in the amygdala differentially modulate nocifensive and affective pain behaviors. Neuropharmacology.

[b0215] Passard A., Attal N., Benadhira R., Brasseur L., Saba G., Sichere P., Perrot S., Januel D., Bouhassira D. (2007). Effects of unilateral repetitive transcranial magnetic stimulation of the motor cortex on chronic widespread pain in fibromyalgia. Brain.

[b0220] Phan K.L., Fitzgerald D.A., Nathan P.J., Moore G.J., Uhde T.W., Tancer M.E. (2005). Neural substrates for voluntary suppression of negative affect: A functional magnetic resonance imaging study. Biol. Psychiatry.

[b0225] Phelps E.A., LeDoux J.E. (2005). Contributions of the Amygdala to Emotion Processing: From Animal Models to Human Behavior. Neuron.

[b0230] Raij T.T., Numminen J., Närvänen S., Hiltunen J., Hari R. (2009). Strength of prefrontal activation predicts intensity of suggestion-induced pain. Hum. Brain Mapp..

[b0235] Robinson M.E., Craggs J.G., Price D.D., Perlstein W.M., Staud R. (2011). Gray Matter Volumes of Pain-Related Brain Areas Are Decreased in Fibromyalgia Syndrome. J. Pain.

[b0240] Rodriguez-Raecke, R., Niemeier, A., Ihle, K., Ruether, W., May, A., 2013. Structural Brain Changes in Chronic Pain Reflect Probably Neither Damage Nor Atrophy. PLOS ONE 8, e54475. https://doi.org/10.1371/journal.pone.0054475. 10.1371/journal.pone.0054475 PMC3566164 23405082

[b0245] Salomons T.V., Johnstone T., Backonja M.-M., Davidson R.J. (2004). Perceived Controllability Modulates the Neural Response to Pain. J. Neurosci..

[b0250] Salomons T.V., Johnstone T., Backonja M.-M., Shackman A.J., Davidson R.J. (2007). Individual Differences in the Effects of Perceived Controllability on Pain Perception: Critical Role of the Prefrontal Cortex. J. Cogn. Neurosci..

[b0255] Seminowicz D.A., Davis K.D. (2006). Cortical responses to pain in healthy individuals depends on pain catastrophizing. Pain.

[b0260] Shuster J., McCormack J., Riddell R.P., Toplak M.E. (2009). Understanding the Psychosocial Profile of Women with Fibromyalgia Syndrome. Pain Res. Manag..

[b0265] Skinner E.A., Zimmer-Gembeck M.J. (2010). Perceived Control and the Development of Coping. Oxford University Press.

[b0275] Tracey I., Mantyh P.W. (2007). The Cerebral Signature for Pain Perception and Its Modulation. Neuron.

[b0280] Tzourio-Mazoyer N., Landeau B., Papathanassiou D., Crivello F., Etard O., Delcroix N., Mazoyer B., Joliot M. (2002). Automated Anatomical Labeling of Activations in SPM Using a Macroscopic Anatomical Parcellation of the MNI MRI Single-Subject Brain. NeuroImage.

[b0285] Valfrè W., Rainero I., Bergui M., Pinessi L. (2007). Voxel-Based Morphometry Reveals Gray Matter Abnormalities in Migraine: January 2008. Headache J. Head Face Pain.

[b0290] Wager T.D., Davidson M.L., Hughes B.L., Lindquist M.A., Ochsner K.N. (2008). Prefrontal-Subcortical Pathways Mediating Successful Emotion Regulation. Neuron.

[b0295] Weisenberg M., Wolf Y., Mittwoch T., Mikulincer M., Aviram O. (1985). Subject versus experimenter control in the reaction to pain. Pain.

[b0305] Whitfield-Gabrieli S., Nieto-Castanon A. (2012). Conn: a functional connectivity toolbox for correlated and anticorrelated brain networks. Brain Connect..

[b0310] Wiech, K., Edwards, R., Moseley, G.L., Berna, C., Ploner, M., Tracey, I., 2014. Dissociable Neural Mechanisms Underlying the Modulation of Pain and Anxiety? An fMRI Pilot Study. PLoS ONE 9, e110654. https://doi.org/10.1371/journal.pone.0110654. 10.1371/journal.pone.0110654 PMC4266493 25502237

[b0315] Wiech K., Seymour B., Kalisch R., Enno Stephan K., Koltzenburg M., Driver J., Dolan R.J. (2005). Modulation of pain processing in hyperalgesia by cognitive demand. NeuroImage.

[b0320] Wiech K., Kalisch R., Weiskopf N., Pleger B., Stephan K.E., Dolan R.J. (2006). Anterolateral prefrontal cortex mediates the analgesic effect of expected and perceived control over pain. J. Neurosci..

[b0325] Wiech K., Farias M., Kahane G., Shackel N., Tiede W., Tracey I. (2008). An fMRI study measuring analgesia enhanced by religion as a belief system. Pain.

[b0330] Wolfe F., Clauw D.J., Fitzcharles M.-A., Goldenberg D.L., Häuser W., Katz R.L., Mease P.J., Russell A.S., Russell I.J., Walitt B. (2016). 2016 Revisions to the 2010/2011 fibromyalgia diagnostic criteria. Semin. Arthritis Rheum..

[b0335] Wu Y.-L., Fang S.-C., Chen S.-C., Tai C.-J., Tsai P.-S. (2021). Effects of Neurofeedback on Fibromyalgia: A Randomized Controlled Trial. Pain Manag. Nurs..

