# Supplementary material for: Neural correlates of control over pain in fibromyalgia patients
Source: Neuroimage Clin. 2023 Feb 21;37:103355. doi: 10.1016/j.nicl.2023.103355 (PMC9982683; doi:10.1016/j.nicl.2023.103355)
Supplement: Supplementary data 1 [file mmc1.docx]

**Supplementary material**

**Psychological Assessment**

FM patients completed the West Haven-Yale Multidimensional Pain Inventory (MPI) (Kerns et al., 1985; German version: Flor et al., 1990), the Fibromyalgia Impact Questionnaire (FIQ-G) (German version: Offenbaecher et al., 2000), the Pain-related Self Statements Scale PRSS (German version: FSS, Flor et al., 1993), the Chronic Pain Grade Scale (CPG) (Von Korff et al., 1992), the Pain Anxiety Symptoms Scale (PASS) (McCracken et al., 1992; German version: Kreddig et al., 2015), the Fear Avoidance Beliefs Questionnaire (FABQ) (Waddell et al., 1993; German version: Pfingsten et al., 2000), the Fibromyalgia Survey Questionnaire (FSQ) (Häuser et al., 2012) and the Pain Perception Scale (Schmerzempfindungsskala, SES) (Heuser and Geissner, 1998).

In patients and controls, presence of depression symptoms was assessed using the Center for Epidemiologic Studies Depression Scale (CES-D; Radloff, 1977; German version: ADS, Hautzinger and Bailer, 1993). Additionally, the Multidimensional Locus of Control IPC Scale (Krampen, 1981) and the Edinburgh Handedness Inventory (EHI) (Oldfield, 1971) were assessed. Table S1 of the supplementary material presents the demographic, psychometric and clinical data for FM patients and HC. The structured clinical interview SKID-I (Wittchen et al., 1997) was conducted with all participants in order to rule out any acute mental illness.

**Clinical and behavioral data analysis**

Clinical data and behavioral measures were analyzed using R Studio Version 1.3.1073 (RStudio Team, 2020) for windows. Age in years, CES-D and IPC scores, individual heat thresholds and the adjusted heat stimulation intensities that were determined prior to the experiment were compared between HC and FM using two sample t tests. In order to compare intensity ratings of the two stimulus conditions, a two-way ANOVA was calculated using group (HC / FM) and controllability (self-controlled heat / computer-controlled heat) as factors. Another two-way ANOVA was conducted in order to investigate the effect of group (HC / FM) and controllability (self-controlled / computer-controlled) on the ratings of perceived control.

| **Table S1**. Demographic, psychometric and clinical data for FM and HC. | | | | | | |
| --- | --- | --- | --- | --- | --- | --- |
|  | FM | | | HC | | |
|  | M | SD | Range | M | SD | Range |
| Age (years) | 50.48 | 9.89 | 32-68 | 46.62 | 13.08 | 28-68 |
| Pain duration in years | 14.88 | 11.82 | 2-44 |  |  |  |
| CES-D* | 22.1 | 6.53 | 14-39 | 6.53 | 14-39 | 6-19 |
| IPC |  |  |  |  |  |  |
| Internality | 34.00 | 4.65 | 22-42 | 36.29 | 4.77 | 24-44 |
| Externality – helplessness* | 23.05 | 6.39 | 10-39 | 19.29 | 4.67 | 13-30 |
| Externality – fatalism* | 24.45 | 6.43 | 10-35 | 21.57 | 3.14 | 18-27 |
| FABQ |  |  |  |  |  |  |
| Fear-avoidance beliefs about work | 15.5 | 11.33 | 0-36 |  |  |  |
| Fear-avoidance beliefs about physical activity | 11.73 | 4.90 | 2-19 |  |  |  |
| FIQ |  |  |  |  |  |  |
| Physical functioning | 1.37 | 0.61 | 0.2-2.4 |  |  |  |
| Total | 222.72 | 234.70 | 18.96-668.72 |  |  |  |
| FSQ |  |  |  |  |  |  |
| Symptom Severity Score | 9.52 | 9.89 | 1-12 |  |  |  |
| Widespread Pain Index | 10.83 | 3.74 | 6-19 |  |  |  |
| PRSS |  |  |  |  |  |  |
| Catastrophizing | 2.30 | 1.19 | 0.00-4.33 |  |  |  |
| Active coping | 3.13 | 0.90 | 0.75-4.88 |  |  |  |
| SES |  |  |  |  |  |  |
| Affective | 35.00 | 10.91 | 15-51 |  |  |  |
| Sensory | 23.17 | 7.22 | 12-39 |  |  |  |
| MPI |  |  |  |  |  |  |
| Pain severity | 4.00 | 15.82 | 6-19 |  |  |  |
| Interference | 4.08 | 1.28 | 0.7-5.7 |  |  |  |
| Life control | 3.44 | 1.34 | 0-6 |  |  |  |
| Affective distress | 3.41 | 1.48 | 0.00-5.67 |  |  |  |
| Support | 4.36 | 1.67 | 0-6 |  |  |  |
| Punishing responses | 1.27 | 1.30 | 0-5.33 |  |  |  |
| Solicitous responses | 3.78 | 1.75 | 0-6 |  |  |  |
| Distracting responses | 3.41 | 1.35 | 0-5.67 |  |  |  |
| General activity level | 7.16 | 2.43 | 2.65-11.45 |  |  |  |

FM: fibromyalgia; HC: healthy controls; M: mean; SD: standard deviation; CES-D: Center for Epidemiologic Studies Depression Scale; IPC: Multidimensional Locus of Control IPC Scale; FABQ: Fear Avoidance Beliefs Questionnaire; FIQ: Fibromyalgia impact questionnaire; FSQ: Fibromyalgia Survey Questionnaire; PRSS: Pain-related Self-statements Scale; SES: Schmerzempfindungsskala; MPI: West Haven-Yale Multidimensional Pain Inventory. * Significant group difference (*p*<.05).

**Clinical data and behavioral effects of perceived control over pain**

Comparing our clinical data between HC and FM, we detected significant differences regarding depressive symptoms (CES-D; *t*(39)=5.69, *p*<.001) as well as the external “helplessness” (*t*(39)=1.99, *p*=.027) and “fatalism” (*t*(39)=1.83, *p*=.038) scales of the IPC, with FM showing higher values in all of these cases (see Table S1).

None of the pain thresholds we obtained were in a pathological/abnormal range. The individual adjusted stimulation intensities that were determined prior to the experiment revealed significant group differences between FM and HC. Accordingly, adjusted stimulation intensities were significantly lower for FM (*M*=45.17°C) than for HC (*M*=47.52°C) (*t*(41)=4.07, *p*<.001, see Table 1). Pain threshold as well as pain tolerance and calculated intensity values did not differ significantly between groups (pain threshold: *t*(41)=1.74, *p*=.089; pain tolerance: *t*(41)=1.7, *p*=.097; calculated intensity: *t*(41)=1.57, *p*=.125).

Examining participants’ intensity ratings, we found significant main effects respectively for group (*F*(1,2152)=72.93, *p*<.001) and controllability (*F*(1,2152)=7.38, *p*=.007), but no significant interaction effect. Ratings of perceived control were higher for HC compared to FM as well as for self-controlled compared to computer-controlled trials. The corresponding ANOVA provided significant main effects for the factor condition (*F*(1,44)=30.2, *p*<.001) and group (*F*(1,44)=5.88, *p*=.02). We could not detect an interaction effect, *F*(1,44)=0.34, *p*=.57.

The adjusted stimulation intensities we used during our experiment were significantly lower for FM compared to HC. This indicates an increased sensitivity to thermal pain in FM. Such changes have been demonstrated numerous times using thermal (Berglund et al., 2002; Cook et al., 2004; Petzke et al., 2003) and other painful stimuli (e.g., cold: Berglund et al., 2002; pressure: Diers et al., 2012; Petzke et al., 2003; electrical: Diers et al., 2008; Sörensen et al., 1998; chemical: Diers et al., 2011; Morris et al., 1998).

In contrast to preceding investigations (Salomons et al., 2004; Wiech et al., 2006), we were not able to detect an analgesic effect of perceived control over pain on pain ratings (Table 1). According to Bräscher et al. (2016), previous brain imaging studies have largely failed to induce behavioral pain-increasing effects of uncontrollable noxious stimuli. In this regard, our findings do correspond to the results of a more recent study by Löffler et al. (Löffler et al., 2018). This investigation found a controllability-induced reduction of pain-related suffering, but no significant changes in perceived pain intensity or unpleasantness. Interestingly, our results are also largely in line with a subsequent study by Wiech et al. (2014) that revealed no relevant analgesic effect of perceived control on average pain ratings, while reporting considerable effects on a neural level. To be more specific, half of the tested participants showed decreased pain intensity ratings following self-controlled compared to computer-controlled trials, but the reverse effect (increased ratings) was observed in the other half. This distribution approximately resembles the pattern we found in our sample, where 27% (HC) / 26% (FM) showed the anticipated effect, 36% (HC) / 43% (FM) showed a trend towards contrary effects and 36% (HC) / 30% (FM) showed no considerable tendency at all. In both groups, some participants displayed definite analgesic effects on pain ratings, whereas others even reported higher pain intensities during controllable pain trials. Regardless of the direction, the vast majority of our subjects displayed rather small effects, leading us to the conclusion that in our experimental setup controllability did not lead to relevant levels of perceived analgesia.

However, the fact that we found effects on a neural level while not being able to show behavioral differences is hardly remarkable. In accordance with Salomons et al. (2004), the neural findings reported above are likely to reflect cognitive and affective processes that are associated with controllability but may be inconsistent between patients regarding individual pain perception. Differences in neural activity are more than a simple reflection of individual pain perception. In this context, Wiech et al. (2014) have proposed that an analgesic effect on pain ratings due to increased controllability might be specific to a particular subgroup of participants, depending on individual variables, such as the personal locus of control. Pain reduction was only observed in subjects who scored highly on a self-efficacy measure for managing pain. The observed absence of a behavioral analgesic effect might be less relevant, as the main concern of our investigation was to examine FM-specific changes regarding neural pain modulation, rather than to show a maximum pain reduction through perceived control. As proposed by Löffler et al. (2018), the individual experience of pain as well as the behavioral effects due to pain controllability appear to be decisively impacted by a complex interaction of individual control beliefs and instructional context.

| **Table S2***.* HC brain responses to self-controlled compared to computer-controlled heat pain. (Related to Figure 2) | | | | | | | |
| --- | --- | --- | --- | --- | --- | --- | --- |
|  |  |  | MNI coordinates | | |  |  |
| Brain region | Laterality | BA | *x* | *y* | *z* | Cluster size^a^ | *Z* score |
| Rolandic operculum | L |  | -46 | -2 | 6 | 73 | 4.16 |
|  | R | 4 | 39 | -4 | 15 | 390 | 3.83 |
| Supramarginal gyrus | L | 40 | -53 | -36 | 27 | 244 | 3.97 |
| OFC | L |  | -25 | 44 | -15 | 20 | 3.94 |
|  | L |  | -7 | 40 | -10 | 38 | 3.91 |
|  | L |  | -23 | 24 | -15 | 23 | 3.36 |
| SMA | L | 6 | -5 | -9 | 59 | 209 | 3.65 |
|  | L | 6 | -37 | -20 | 63 | 32 | 2.99 |
|  | R | 6 | 7 | 5 | 73 | 15 | 3.11 |
| Caudate nucleus | L |  | -19 | 17 | 6 | 35 | 3.61 |
|  | R |  | 18 | 19 | 8 | 17 | 3.43 |
| Cerebellum | R | 19 | 21 | -57 | -29 | 18 | 3.5 |
| Thalamus | R |  | 4 | -18 | -3 | 28 | 3.47 |
| Insula | L | 13 | -37 | -13 | -6 | 23 | 3.46 |
|  | L | 13 | -37 | 10 | -6 | 9 | 3.4 |
|  | L | 13 | -28 | 26 | 6 | 23 | 3.03 |
|  | R | 13 | 32 | -22 | 13 | 22 | 3.19 |
| VLPFC | R | 10 | 34 | 42 | 22 | 30 | 3.36 |
|  | R | 46 | 46 | 44 | 10 | 7 | 2.62 |
|  | L | 46 | -35 | 49 | 24 | 17 | 3.06 |
| dACC | R | 32 | 11 | 35 | 22 | 20 | 3.19 |
|  | R | 32 | 9 | 10 | 40 | 25 | 2.88 |
|  | R | 32 | 4 | 21 | 43 | 2 | 2.58 |
|  | L | 32 | -5 | 8 | 45 | 37 | 3.18 |
|  | L | 32 | -12 | 21 | 40 | 7 | 3.12 |
| DLPFC | R | 9 | 27 | 49 | 31 | 8 | 3.04 |
|  | R | 9 | 25 | 42 | 24 | 6 | 2.85 |

MNI: Montreal Neurological Institute; BA: Brodmann area; L: left; R: right; OFC: orbitofrontal cortex; SMA: supplementary motor area; VLPFC: ventrolateral prefrontal cortex; dACC: dorsal anterior cingulate cortex; DLPFC: dorsolateral prefrontal cortex. *p* ≤ .001; voxel size: 2.3 x 2.3 x 2.3 mm; ^a^ in voxel.

| **Table S3**. Group contrast (HC > FM) of brain responses for self-controlled compared to computer-controlled heat pain. (Related to Figure 3) | | | | | | | |
| --- | --- | --- | --- | --- | --- | --- | --- |
|  |  |  | MNI coordinates | | |  |  |
| Brain region | Laterality | BA | *x* | *y* | *z* | Cluster size^a^ | *Z* score |
| Angular gyrus | L | 39 | -55 | -55 | 20 | 19 | 3.94 |
|  | R | 39 | 39 | -66 | 54 |  |  |
| Superior temporal gyrus | R | 48 | 60 | -18 | 8 | 13 | 3.88 |
| Superior parietal lobule | R | 7 | 16 | -75 | 52 | 29 | 3.61 |
|  | L | 7 | -30 | -59 | 63 | 6 | 3.24 |
| Putamen | R |  | 25 | 1 | 1 | 3 | 3.27 |
|  | R |  | 30 | 1 | 1 | 1 | 2.7 |
| Hypothalamus | R |  | 0 | -4 | -10 | 23 | 3.06 |
| Precuneus | L |  | -5 | -66 | 61 | 3 | 3.02 |
|  | R |  | 9 | -64 | 66 | 3 | 2.95 |
| Caudate nucleus | R |  | 16 | 19 | 8 | 5 | 2.95 |
| VLPFC | R |  | 37 | 37 | 17 | 5 | 2.86 |
| Opercular IFG | L |  | -51 | 14 | 33 | 10 | 2.82 |
| Amygdala | L |  | -19 | 1 | -17 | 4 | 2.81 |
| MFG | L |  | -39 | 24 | 50 | 2 | 2.76 |
| Superior frontal gyrus | L | 10 | -14 | 67 | 1 | 2 | 2.74 |
| SMA | R/L | 6 | 0 | -13 | 63 | 8 | 2.69 |
| DLPFC | R | 9 | 11 | 35 | 22 | 4 | 2.64 |

MNI: Montreal Neurological Institute; BA: Brodmann area; L: left; R: right; VLPFC: ventrolateral prefrontal cortex; IFG: inferior frontal gyrus; MFG: middle frontal gyrus; SMA: supplementary motor area; DLPFC: dorsolateral prefrontal cortex. *p*<.005; voxel size: 2.3 x 2.3 x 2.3 mm; ^a^ in voxel.

**REFERENCES**

Berglund, B., Harju, E.-L., Kosek, E., Lindblom, U., 2002. Quantitative and qualitative perceptual analysis of cold dysesthesia and hyperalgesia in fibromyalgia. Pain 96, 177–187. https://doi.org/10.1016/S0304-3959(01)00443-2

Bräscher, A.-K., Becker, S., Hoeppli, M.-E., Schweinhardt, P., 2016. Different Brain Circuitries Mediating Controllable and Uncontrollable Pain. J. Neurosci. 36, 5013–5025. https://doi.org/10.1523/JNEUROSCI.1954-15.2016

Cook, D.B., Lange, G., Ciccone, D.S., Wen-Ching, L., Steffener, J., Natelson, B.H., 2004. Functional imaging of pain in patients with primary fibromyalgia. J. Rheumatol. 31, 364–378.

Diers, M., Koeppe, C., Yilmaz, P., Thieme, K., Markela-Lerenc, J., Schiltenwolf, M., van Ackern, K., Flor, H., 2008. Pain ratings and somatosensory evoked responses to repetitive intramuscular and intracutaneous stimulation in fibromyalgia syndrome. J. Clin. Neurophysiol. 25, 153–160. https://doi.org/10.1097/WNP.0b013e31817759c5

Diers, M., Schley, M.T., Rance, M., Yilmaz, P., Lauer, L., Rukwied, R., Schmelz, M., Flor, H., 2011. Differential central pain processing following repetitive intramuscular proton/prostaglandin E2 injections in female fibromyalgia patients and healthy controls. Eur. J. Pain 15, 716–723. https://doi.org/10.1016/j.ejpain.2010.12.002

Diers, M., Yilmaz, P., Rance, M., Thieme, K., Gracely, R.H., Rolko, C., Schley, M.T., Kiessling, U., Wang, H., Flor, H., 2012. Treatment-related changes in brain activation in patients with fibromyalgia syndrome. Exp. Brain Res. 218, 619–628. https://doi.org/10.1007/s00221-012-3055-2

Flor, H., Behle, D.J., Birbaumer, N., 1993. Assessment of pain-related cognitions in chronic pain patients. Behav. Res. Ther. 31, 63–73. https://doi.org/10.1016/0005-7967(93)90044-U

Flor, H., Rudy, T.E., Birbaumer, N., Streit, B., Schugens, M.M., 1990. Zur Anwendbarkeit des West Haven-Yale Multidimensional Pain Inventory im deutschen Sprachraum. Schmerz 4, 82–87. https://doi.org/10.1007/BF02527839

Häuser, W., Jung, E., Erbslöh-Möller, B., Gesmann, M., Kühn-Becker, H., Petermann, F., Langhorst, J., Weiss, T., Winkelmann, A., Wolfe, F., 2012. Validation of the Fibromyalgia Survey Questionnaire within a Cross-Sectional Survey. PLoS ONE 7, e37504. https://doi.org/10.1371/journal.pone.0037504

Hautzinger, M., Bailer, M., 1993. Allgemein Depressionsskala (ADS) [General Depression Scale]. Beltz Test.

Heuser, J., Geissner, E., 1998. Computer-Version der Schmerzempfindungsskala SES. Schmerz 12, 205–208. https://doi.org/10.1007/s004820050143

Kerns, R.D., Turk, D.C., Rudy, T.E., 1985. The West Haven-Yale Multidimensional Pain Inventory (WHYMPI). Pain 23, 345–356. https://doi.org/10.1016/0304-3959(85)90004-1

Krampen, G., 1981. IPC-Fragebogen zu Kontrollüberzeugungen.

Kreddig, N., Rusu, A.C., Burkhardt, K., Hasenbring, M.I., 2015. The German PASS-20 in Patients with Low Back Pain: New Aspects of Convergent, Divergent, and Criterion-Related Validity. Int. J. Behav. Med. 22, 197–205. https://doi.org/10.1007/s12529-014-9426-2

Löffler, M., Kamping, S., Brunner, M., Bustan, S., Kleinböhl, D., Anton, F., Flor, H., 2018. Impact of controllability on pain and suffering. PAIN Rep. 3, e694. https://doi.org/10.1097/PR9.0000000000000694

McCracken, L.M., Zayfert, C., Gross, R.T., 1992. The Pain Anxiety Symptoms Scale: development and validation of a scale to measure fear of pain. Pain 50, 67–73. https://doi.org/10.1016/0304-3959(92)90113-P

Morris, V., Cruwys, S., Kidd, B., 1998. Increased capsaicin-induced secondary hyperalgesia as a marker of abnormal sensory activity in patients with fibromyalgia. Neurosci. Lett. 250, 205–207. https://doi.org/10.1016/S0304-3940(98)00443-1

Offenbaecher, M., Waltz, M., Schoeps, P., 2000. Validation of a German version of the Fibromyalgia Impact Questionnaire (FIQ-G). J. Rheumatol. 27, 1984–1988.

Oldfield, R.C., 1971. The assessment and analysis of handedness: The Edinburgh Inventory. Neuropsychologia 9, 97–113. https://doi.org/10.1016/0028-3932(71)90067-4

Petzke, F., Clauw, D.J., Ambrose, K., Khine, A., Gracely, R.H., 2003. Increased pain sensitivity in fibromyalgia: effects of stimulus type and mode of presentation. Pain 105, 403–413. https://doi.org/10.1016/S0304-3959(03)00204-5

Pfingsten, M., Kröner-Herwig, B., Leibing, E., Kronshage, U., Hildebrandt, J., 2000. Validation of the German version of the Fear-Avoidance Beliefs Questionnaire (FABQ). Eur. J. Pain 4, 259–266. https://doi.org/10.1053/eujp.2000.0178

Radloff, L.S., 1977. The CES-D Scale: A Self-Report Depression Scale for Research in the General Population. Appl. Psychol. Meas. 1, 385–401. https://doi.org/10.1177/014662167700100306

Salomons, T.V., Johnstone, T., Backonja, M.-M., Davidson, R.J., 2004. Perceived Controllability Modulates the Neural Response to Pain. J. Neurosci. 24, 7199–7203. https://doi.org/10.1523/JNEUROSCI.1315-04.2004

Sörensen, J., Graven-Nielsen, T., Henriksson, K.G., Bengtsson, Arendt-Nielsen, L., 1998. Hyperexcitability in Fibromyalgia. J. Rheumatol. 25, 152–155.

Von Korff, M., Ormel, J., Keefe, F.J., Dworkin, S.F., 1992. Grading the severity of chronic pain. Pain 50, 133–149. https://doi.org/10.1016/0304-3959(92)90154-4

Waddell, G., Newton, M., Henderson, I., Somerville, D., Main, C.J., 1993. A Fear-Avoidance Beliefs Questionnaire (FABQ) and the role of fear-avoidance beliefs in chronic low back pain and disability. Pain 52, 157–168. https://doi.org/10.1016/0304-3959(93)90127-B

Wiech, K., Edwards, R., Moseley, G.L., Berna, C., Ploner, M., Tracey, I., 2014. Dissociable Neural Mechanisms Underlying the Modulation of Pain and Anxiety? An fMRI Pilot Study. PLoS ONE 9, e110654. https://doi.org/10.1371/journal.pone.0110654

Wiech, K., Kalisch, R., Weiskopf, N., Pleger, B., Stephan, K.E., Dolan, R.J., 2006. Anterolateral prefrontal cortex mediates the analgesic effect of expected and perceived control over pain. J. Neurosci. 26, 11501–11509. https://doi.org/10.1523/JNEUROSCI.2568-06.2006

Wittchen, H.U., Wunderlich, U., Gruschwitz, S., Zaudig, M., 1997. SKID I. Strukturiertes Klinisches Interview für DSM-IV. Achse I: Psychische Störungen. Interviewheft und Beurteilungsheft. Eine deutschsprachige, erweiterte Bearb. d. amerikanischen Originalversion des SKID I.
